# SCIseg: Automatic Segmentation of Intramedullary Lesions in Spinal Cord Injury on T2-weighted MRI Scans

**DOI:** 10.1148/ryai.240005

**Published:** 2024-11-06

**Authors:** Enamundram Naga Karthik, Jan Valošek, Andrew C. Smith, Dario Pfyffer, Simon Schading-Sassenhausen, Lynn Farner, Kenneth A. Weber, Patrick Freund, Julien Cohen-Adad

**Affiliations:** From the NeuroPoly Laboratory, Institute of Biomedical Engineering, Polytechnique Montréal, 2500 Chemin de Polytechnique, Montréal, Québec, Canada H3T 1J4 (E.N.K., J.V., J.C.A.); Mila-Quebec AI Institute, Montréal, Québec, Canada (E.N.K., J.V., J.C.A.); Department of Neurosurgery and Department of Neurology, Faculty of Medicine and Dentistry, Palacký University Olomouc, Olomouc, Czechia (J.V.); Department of Physical Medicine and Rehabilitation Physical Therapy Program, University of Colorado School of Medicine, Aurora, Colo (A.C.S.); Spinal Cord Injury Center, Balgrist University Hospital, University of Zürich, Zürich, Switzerland (D.P., S.S.S., L.F., P.F.); Department of Anesthesiology, Perioperative and Pain Medicine, Stanford University School of Medicine, Stanford, Calif (D.P., K.A.W.); Department of Neurophysics, Max Planck Institute for Human Cognitive and Brain Sciences, Leipzig, Germany (P.F.); and Functional Neuroimaging Unit, CRIUGM and Centre de Recherche du CHU Sainte-Justine, Université de Montréal, Montréal, Québec, Canada (J.C.A.).

**Keywords:** Spinal Cord, Trauma, Segmentation, MR Imaging, Supervised Learning, Convolutional Neural Network (CNN)

## Abstract

**Purpose:**

To develop a deep learning tool for the automatic segmentation of the spinal cord and intramedullary lesions in spinal cord injury (SCI) on T2-weighted MRI scans.

**Materials and Methods:**

This retrospective study included MRI data acquired between July 2002 and February 2023. The data consisted of T2-weighted MRI scans acquired using different scanner manufacturers with various image resolutions (isotropic and anisotropic) and orientations (axial and sagittal). Patients had different lesion etiologies (traumatic, ischemic, and hemorrhagic) and lesion locations across the cervical, thoracic, and lumbar spine. A deep learning model, SCIseg (which is open source and accessible through the Spinal Cord Toolbox, version 6.2 and above), was trained in a three-phase process involving active learning for the automatic segmentation of intramedullary SCI lesions and the spinal cord. The segmentations from the proposed model were visually and quantitatively compared with those from three other open-source methods (PropSeg, DeepSeg, and contrast-agnostic, all part of the Spinal Cord Toolbox). The Wilcoxon signed rank test was used to compare quantitative MRI biomarkers of SCI (lesion volume, lesion length, and maximal axial damage ratio) derived from the manual reference standard lesion masks and biomarkers obtained automatically with SCIseg segmentations.

**Results:**

The study included 191 patients with SCI (mean age, 48.1 years ± 17.9 [SD]; 142 [74%] male patients). SCIseg achieved a mean Dice score of 0.92 ± 0.07 and 0.61 ± 0.27 for spinal cord and SCI lesion segmentation, respectively. There was no evidence of a difference between lesion length (*P* = .42) and maximal axial damage ratio (*P* = .16) computed from manually annotated lesions and the lesion segmentations obtained using SCIseg.

**Conclusion:**

SCIseg accurately segmented intramedullary lesions on a diverse dataset of T2-weighted MRI scans and automatically extracted clinically relevant lesion characteristics.

**Keywords:** Spinal Cord, Trauma, Segmentation, MR Imaging, Supervised Learning, Convolutional Neural Network (CNN)

Published under a CC BY 4.0 license.

## Graphical abstract

**Figure:**
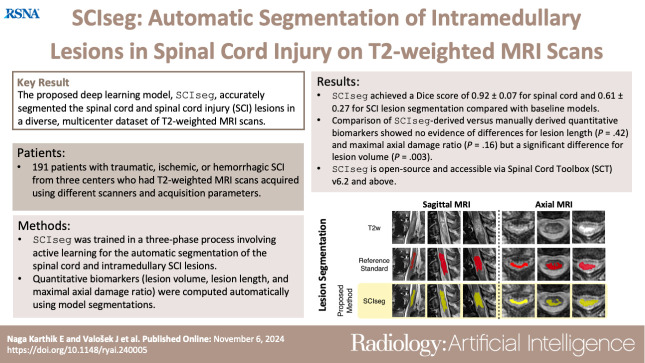

**Summary**

The proposed deep learning model accurately segmented the spinal cord and spinal cord injury lesions in a diverse, multicenter dataset of T2-weighted MRI scans.

**Key Points**

■ The SCIseg deep learning model was developed for segmentation of the spinal cord and intramedullary lesions using a multicenter dataset of 191 patients with spinal cord injury who underwent T2-weighted MRI examinations using various scanner manufacturers and acquisition parameters.
■ SCIseg achieved a mean Dice score of 0.92 ± 0.07 (SD) and 0.61 ± 0.27 for spinal cord and spinal cord injury lesion segmentation, respectively.
■ There was no evidence of a difference between quantitative MRI biomarkers, namely, lesion length (*P* = .42) and maximal axial damage ratio (*P* = .16), computed from manually annotated lesions and the lesion segmentations obtained using SCIseg.

## Introduction

Spinal cord injury (SCI) refers to damage to the spinal cord due to traumatic or nontraumatic processes. Traumatic SCI results from acute damage to the spinal cord due to external physical factors (1,2). Most patients with traumatic SCI sustain permanent neurologic deficits such as motor and autonomic dysfunction, with devastating physical and social consequences (1). Degenerative cervical myelopathy (DCM), the most common form of nontraumatic SCI, originates from chronic mechanical compression of the spinal cord (3). Although relatively less common than traumatic lesions, ischemic SCI lesions represent up to 20% of all nontraumatic lesions (4,5) and show a similar course of recovery to traumatic SCI (6,7).

MRI scans of patients with SCI provide macrostructural information about the level of injury and intramedullary abnormalities (eg, edema and hemorrhage) and allow for the evaluation of soft tissue structures (1,3). Importantly, MRI-derived quantitative biomarkers, such as intramedullary lesion length and lesion volume, have demonstrated associations with the neurologic prognosis of patients with traumatic SCI (7–12). Particularly, smaller lesion length and area were significantly associated with better recovery of patients (7,9,10).

Despite recent advances in the automatic processing of spinal cord MRI (13–16), robust methods for detecting quantitative MRI biomarkers in SCI are lacking. As a result, most studies involve manual identification of these biomarkers (7,11,17–20). This task is time-consuming and further hampered by interrater variability, making it impractical in clinical trials (3). Furthermore, segmentation of intramedullary SCI lesions on MRI scans poses an extremely challenging task mainly due to the evolving appearance of lesions in different injury phases (eg, acute, subacute, intermediate) (1,2). Surgical implants in postoperative MRI scans might also cause severe image artifacts. Deep learning (DL) can improve diagnosis and prognostication in SCI by automating the lesion annotation process, thereby reducing rater-specific biases and facilitating the analysis of large SCI cohorts across sites (21–23). Indeed, quantitative SCI lesion biomarkers derived from DL-based automatic segmentations have been shown to correlate well with clinical measures of motor impairment (24). Despite its numerous potential advantages, DL has not been sufficiently explored in the context of SCI (22), with no open-source methods existing to date. This gap suggests the need for an automatic biomarker identification method that takes into account the complex pathophysiology of patients with SCI, generalizes to multiple sites, and remains easily accessible by researchers.

The purpose of this study was to develop an open-source DL-based tool, SCIseg, for the automatic segmentation of the spinal cord and intramedullary lesions on T2-weighted MRI scans of patients with SCI. Model-derived quantitative MRI biomarkers of SCI, such as lesion volume, lesion length, and maximal axial damage ratio, were compared with biomarkers derived from manual reference standard. Furthermore, correlation analyses were conducted between biomarkers derived using the automatic SCIseg and clinical scores, specifically light touch, pinprick, and lower extremity motor scores.

## Materials and Methods

### Study Design and Patients

This retrospective study included 191 patients with SCI who underwent MRI at three sites (Balgrist University Hospital Zürich, Zürich, Switzerland [site 1]; Craig Hospital, Englewood, Colorado [site 2]; Pitié-Salpêtrière University Hospital, Paris, France [site 3]) between July 2002 and February 2023. All patients provided written informed consent following institutional review board approval and the Declaration of Helsinki. The inclusion criteria were traumatic, ischemic, or hemorrhagic SCI; presence or absence of surgical hardware; and clinical data available for analyses. Exclusion criteria were concurrent traumatic brain injury beyond concussion and significant preexisting neurologic history (ie, multiple sclerosis, transverse myelitis, cerebrovascular stroke). Patients from site 2 were clinically assessed using the international standards for the neurologic classification of SCI protocol (25) to obtain light touch, pinprick, and lower extremity motor scores, as described by Smith et al (11,26). All 191 patients from the three sites were reported previously (7,11,17,26,27). These articles used manually annotated lesion masks to study the clinical consequences of SCI and their predictive relationships with motor and sensory functions. In contrast, our study presents a DL-based tool to segment intramedullary SCI lesions automatically.

### MRI Data and Reference Standard

**Figure 1:**
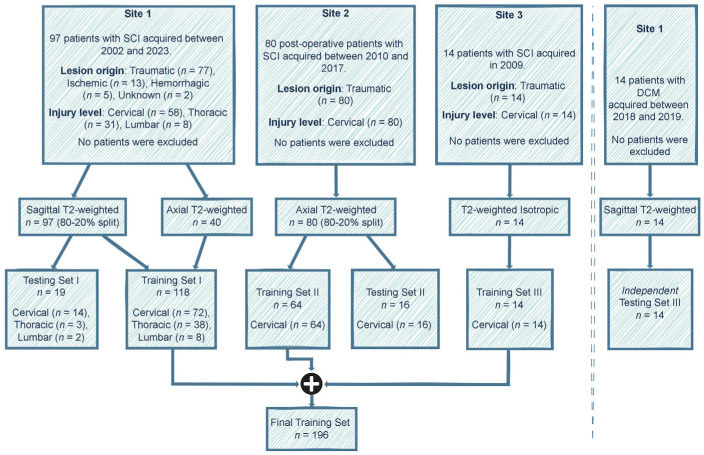
Study flowchart. The data included patient cohorts from three sites with heterogeneous image resolutions, orientations, and lesion etiologies. The validation set is included within the final training set. Models were evaluated independently on the test sets of site 1 and site 2, along with their evaluation on an external test set of patients with degenerative cervical myelopathy (DCM). See Table 1 for details on the MRI vendors and field strengths. SCI = spinal cord injury.

The MRI scans were converted from Digital Imaging and Communications in Medicine (DICOM) to Neuroimaging Informatics Technology Initiative (NIfTI) format and organized according to the Brain Imaging Data Structure standard (28) at individual sites. During this curation process, all sensitive patient information was deleted. T2-weighted MRI scans with varying lesion etiologies (traumatic, ischemic, hemorrhagic), injury chronicity (subacute, intermediate, and chronic), orientations (sagittal and axial), and voxel sizes were used for this study (Fig 1, Table 1). Lesions appearing as T2-weighted signal abnormalities (hyperintense or hypointense voxels corresponding to primary contusions, secondary cytotoxic edema, or hemorrhage) were manually annotated as a single object by two raters from site 1 (D.P. and L.F.), one rater from site 2 (A.C.S.), and by two raters (E.N.K. and J.V.) for site 3 using JIM (version 7.0; Xinapse Systems) and FSLeyes (version 1.9.0; University of Oxford) image viewers. As obtaining the reference standard spinal cord segmentation masks using a fully manual approach is time-consuming, sct_deepseg_sc (29) was used to initially segment the spinal cord of patients from all three sites, followed by manual corrections wherever necessary. This semiautomatic approach was also used in previous studies (29,30).

**Table 1:**
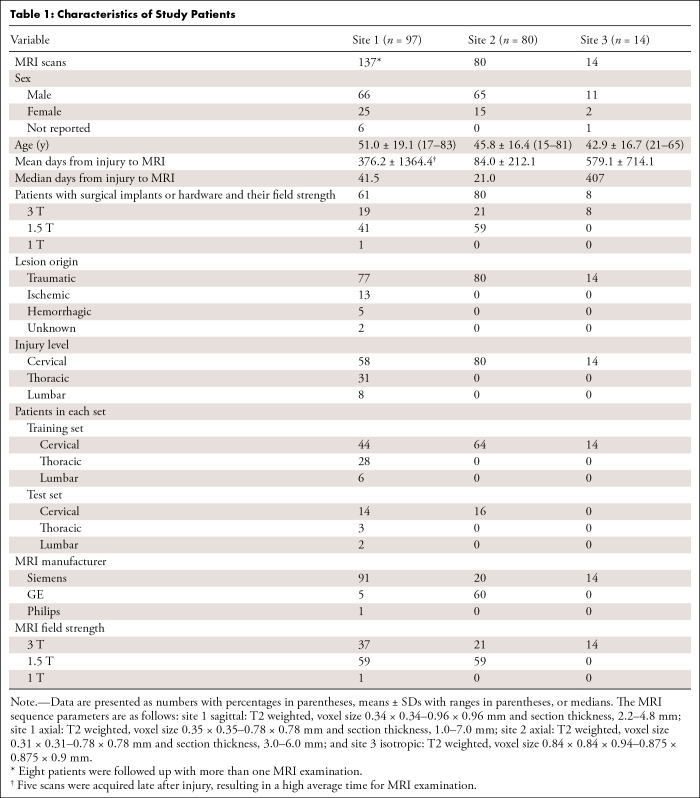
Characteristics of Study Patients

### DL Training Protocol

**Figure 2:**
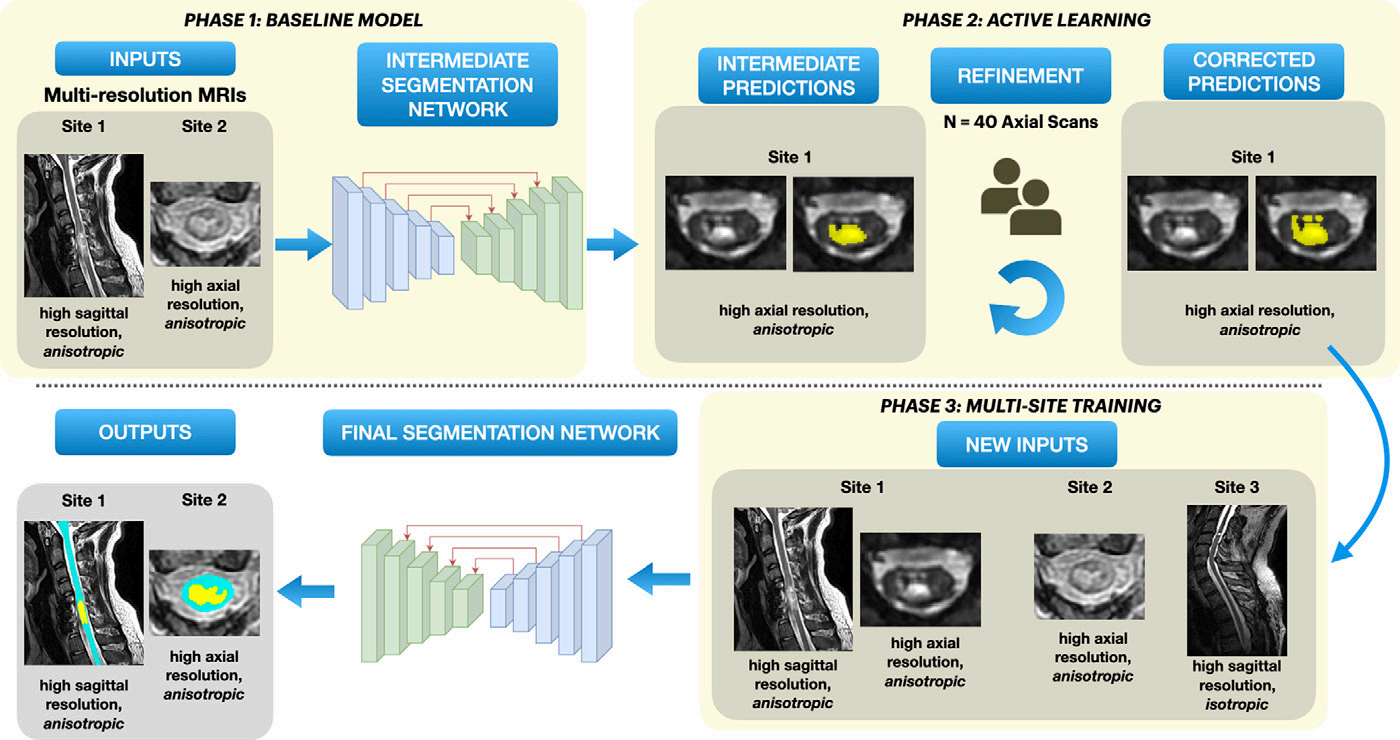
Overview of our segmentation approach. Phase 1: A baseline model is trained on data consisting of T2-weighted scans with axial and sagittal orientations from two sites. Phase 2: Active learning. Initial batch of automatic predictions on T2-weighted axial scans from site 2 are obtained, followed by manual corrections. Phase 3: Along with the newly corrected axial scans, isotropic T2-weighted sagittal scans from site 3 are added to the original dataset for multisite training. The final model is trained to segment both spinal cord and lesion simultaneously.

The model was trained in three phases (Fig 2). In the initial phase, a baseline segmentation model was trained using a labeled dataset of 78 patients with T2-weighted sagittal scans (site 1) and 64 patients with T2-weighted axial scans (site 2). We used the region-based training strategy of nnU-Net (31), in which the model learns to segment the spinal cord and the lesions simultaneously. The lesions are segmented as a single object covering hyperintense and hypointense voxels, hence containing both edema and hemorrhage. The following default nnU-Net data augmentation methods were used: random rotation, scaling, mirroring, Gaussian noise addition, Gaussian blurring, image brightness and contrast adjustment, low-resolution simulation, and gamma transformation. All scans were preprocessed in right, posterior, inferior orientation, resampled to a common resolution (0.78 × 0.56 × 0.78 mm, corresponding to the median of all image resolutions in the training set), and intensity normalized using *z* score normalization. The model was trained for 1000 epochs, with a batch size of two using the stochastic gradient descent optimizer with a polynomial learning rate scheduler.

For the second phase, we used the human-in-the-loop active learning strategy (32) to gradually include axial T2-weighted scans from site 1 in the training dataset. Using the phase 1 baseline model, we generated initial spinal cord and lesion predictions for unlabeled axial scans from site 1. In a subset of 40 scans, predicted segmentations underwent quality control, with two raters (E.N.K. and J.V.) manually correcting if needed. These refined segmentations were then added to the training dataset, resulting in 182 scans in the training set.

For the third training phase, to further improve our model’s generalization capabilities to a wide range of image resolutions, we added a new dataset from site 3 containing 14 isotropic resolution T2-weighted sagittal scans of patients with traumatic SCI.

The final dataset consisting of 196 scans from three sites was used for training the model with the region-based strategy described above.

### Evaluation Protocol

We created two independent test sets (site 1: *n* = 19; site 2: *n* = 16), following the 80/20 train/test splitting ratio (Table 1). To ensure an unbiased assessment of the model’s performance and avoid overfitting, the train/test splits were done at the patient level rather than the image level, meaning that for the subset of 40 patients with sagittal and axial scans, both scans were included in the training split. For the test set, however, only the sagittal scans of each patient were used. We trained five models, each with a different train/test split using a different random seed, to avoid biasing the model toward a particular dataset split. We chose to use five random seeds instead of one fivefold cross-validation to increase the likelihood of more diverse test sets containing patients with SCI with well-defined hyperintense lesions and lesions appearing under severe metal implants. The model’s performance in spinal cord segmentation was evaluated independently within each test set by comparing it with open-source methods available in the Spinal Cord Toolbox (SCT) (15): sct_propseg (33), sct_deepseg_sc (29), and the recently proposed contrast-agnostic spinal cord segmentation model (30). Due to the lack of existing state-of-the-art, open-source methods for SCI lesion segmentation, we compared the three-dimensional (3D) version of the SCIseg model with its two-dimensional (2D) version. Additionally, we tested our model on an independent cohort of 14 patients with DCM from site 1 (unseen during training) to evaluate its generalization on patients with nontraumatic SCI (Fig 1).

### Evaluation Metrics

For quantitative evaluation of model performance, we used the segmentation metrics from the open-source Anima toolkit (*https://anima.readthedocs.io/en/latest/index.html*). For spinal cord segmentation, we calculated the Dice score and the relative volume error (RVE). For lesion segmentation, we calculated the Dice score, average surface distance, lesion-wise positive predictive value, lesion-wise sensitivity, and F1 score (34). As we trained five models on five random train/test splits (instead of fivefold cross-validation), some patients were present in more than one test set. We thus averaged the metrics across test splits for such patients.

### Quantitative MRI Biomarkers

We used the Spinal Cord Toolbox’s sct_analyze_lesion function to automatically compute the total lesion volume, intramedullary lesion length, and maximal axial damage ratio (11) from the manual reference standard lesion masks and the automatic predictions using the proposed SCIseg 3D model. To assess the effect of adding more training data during active learning, we computed the quantitative MRI biomarkers before (phase 1) and after active learning (phase 3). The quantitative MRI biomarkers were then averaged across five random test splits. Additionally, for site 2, we correlated the quantitative MRI biomarkers with the clinical scores (light touch, pinprick, and lower extremity motor scores).

### Statistical Analysis

Statistical analysis was performed using the SciPy Python library, version 1.11.4 (35). Data normality was tested using the D’Agostino and Pearson normality test. Within-site comparisons of age and sex between patients from the testing and training sets were performed using the Mann-Whitney *U* test and the χ^2^ test, respectively. Between-group comparisons (spinal cord segmentation performance SCIseg vs open-source methods; lesion segmentation performance SCIseg 2D vs SCIseg 3D; SCIseg lesion segmentation performance before [phase 1] vs after [phase 3] active learning; manual reference standard lesion masks vs SCIseg-predicted lesions) were performed using the Wilcoxon signed rank test. The SCIseg lesion segmentation performance between sites 1 and 2 was tested using the Mann-Whitney *U* test. Correlations between clinical scores and quantitative MRI biomarkers were examined using the Spearman rank-order correlation. *P* < .05 was considered to indicate a statistically significant difference.

### Code Availability

Following open science principles and to facilitate reproducibility, all codes, processing scripts, and results are open source and freely available to the SCI research community at *https://github.com/ivadomed/model_seg_sci/releases/tag/r20240716*.

## Results

### Patient Characteristics

A total of 191 patients (mean age, 48.1 years ± 17.9 [SD]; 142 males, 42 [22%] females, seven [4%] sex not reported) with 231 MRI scans from three sites with different lesion etiologies (traumatic, ischemic, and hemorrhagic) were included in this study (Fig 1). Ninety-seven patients were from site 1, of which 61 had surgical hardware (dorsal or ventral spondylodesis), 13 underwent decompressive surgery, and 23 did not undergo surgery. Eighty patients were from site 2, all of which had postoperative metallic stabilization. Fourteen patients were from site 3, of which eight had surgical hardware, two had decompressive surgery, and four did not undergo any surgery. Details about patient demographics, injury levels, injury chronicity, and scanner types can be found in Table 1.

Eight patients from site 1 were followed up with additional MRI examinations. The final training set comprised 196 MRI scans, and the final testing set contained 35 MRI scans. There was no evidence of within-site differences in age (site 1: *P* = .06; site 2: *P* = .20) and sex (site 1: *P* = .71; site 2: *P* = .72) between patients from the testing and training sets. Patients were scanned across scanners from different manufacturers (Siemens, GE, Philips) with different field strengths (1 T, 1.5 T, 3 T). T2-weighted scans used in this study had heterogeneous image resolutions and orientations (Table 1).

### Automatic Spinal Cord and Lesion Segmentation in SCI

Table 2 shows the quantitative results of SCIseg 3D on test sets of the two sites stratified by scanner strength along with their averages. As shown by the Dice scores, spinal cord segmentations from the model were stable across different data splits and magnetic field strengths despite the presence of MRI artifacts induced by spinal hardware on the scans. However, for lesion segmentation, the model performed better on site 2 compared with site 1 (mean Dice score for site 2, 0.74 ± 0.15 vs site 1, 0.51 ± 0.30), with a high SD across splits.

**Table 2:**
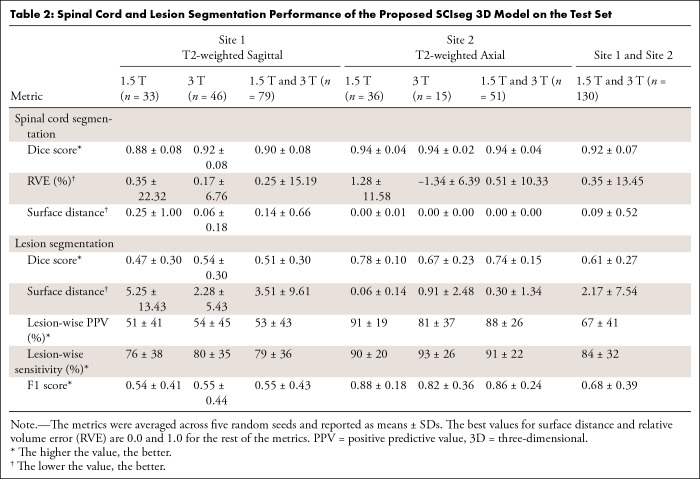
Spinal Cord and Lesion Segmentation Performance of the Proposed SCIseg 3D Model on the Test Set

### Comparison with Other Methods

**Figure 3:**
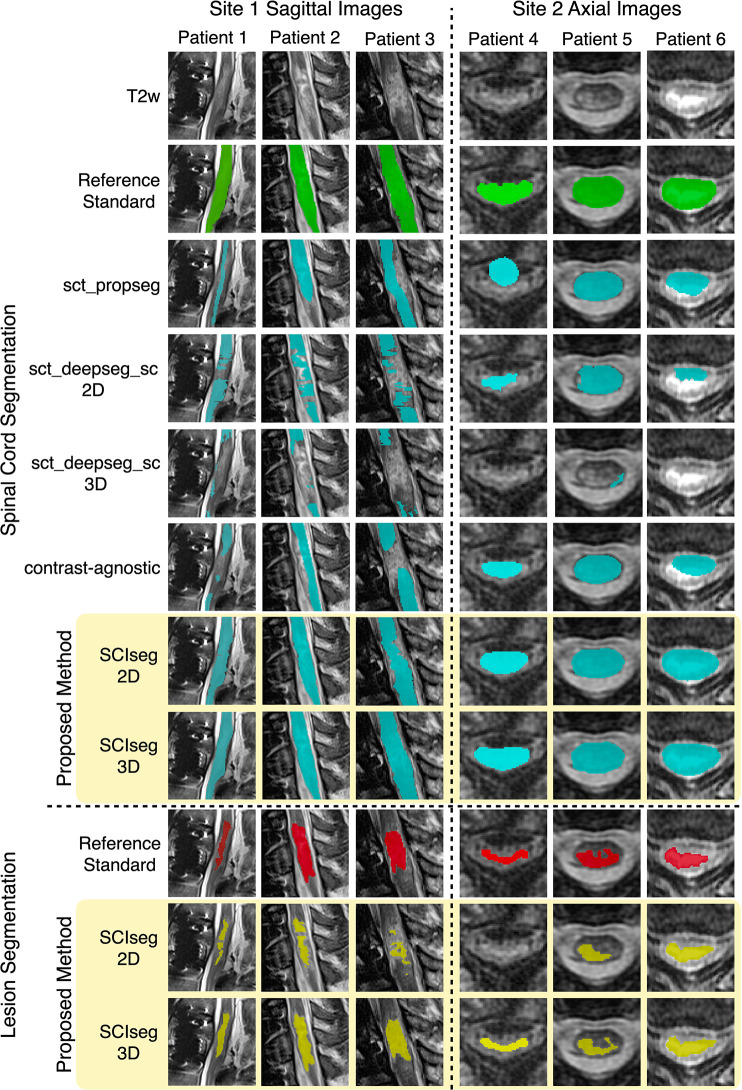
Comparison of SCIseg with baseline methods for the spinal cord and lesion segmentation in patients from site 1 and site 2. SCIseg 3D provides the best qualitative results for both spinal cord and lesion segmentation. Green and teal colors refer to the reference standard and automatic predictions for the spinal cord. Red and yellow refer to the reference standard and the automatic predictions for lesions. 3D = three-dimensional, 2D = two-dimensional, T2w = T2-weighted.

**Figure 4:**
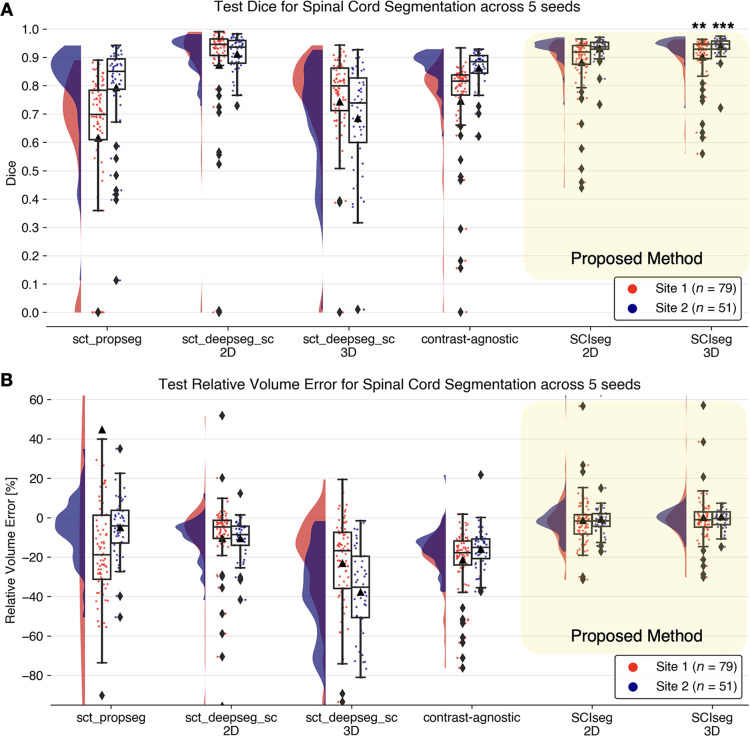
Raincloud plots compare the **(A)** Dice scores (best: 1; worst: 0) and **(B)** relative volume error (best: 0%) across various spinal cord segmentation methods. The numbers in the legend represent the number of test scans in each site across five different training seeds. Although the sct_deepseg_sc 2D and SCIseg 3D have similar Dice scores, the former shows a higher undersegmentation (negative relative volume error) compared with the latter. ** *P* < .001 (statistically significant for all pairs except SCIseg 3D and sct_deepseg_sc 2D). *** *P* < .05 (two-sided Bonferroni-corrected pairwise Wilcoxon signed rank test for SCIseg 3D with all baselines). 3D = three-dimensional, 2D = two-dimensional.

The comparison of spinal cord and SCI lesion segmentation performance of SCIseg 3D with other methods is shown in Figures 3 and 4. The half-violin plots in Figure 4 show the distribution of the Dice scores and RVE for test scans across all seeds, and the scatterplots show the performance of the models on each test scan.

***Spinal cord segmentation.—*** SCIseg 3D achieved the best segmentation performance (mean Dice score, 0.92 ± 0.07; mean RVE, 0.35 ± 13.45) for both sites compared with other baselines (Fig 4). For site 1, the Wilcoxon signed rank test revealed significant differences (*P* < .001, Bonferroni corrected) in Dice score between SCIseg 3D and all baseline methods except for sct_deepseg_sc 2D. As described in the MRI Data and Reference Standard section, this finding is the consequence of using a semiautomatic approach involving sct_deepseg_sc 2D to create the reference standard spinal cord segmentation masks. As a result, quantitative evaluations involving the reference standard masks obtained from this baseline model were inherently biased to be higher than the rest of the methods in comparison. Although there was no significant difference between SCIseg 3D and sct_deepseg_sc 2D, the SCIseg 3D model obtained spinal cord segmentations for all test cases, including those in which the baselines obtained empty predictions (shown by diamonds at Dice = 0 in Fig 4A). For site 2, statistically significant differences in Dice scores were found between SCIseg 3D and all baselines (*P* < .05, Bonferroni corrected). We observed more under- and oversegmented predictions by SCIseg 3D for site 1 relative to site 2 (shown by a larger spread of scatter points around RVE = 0% in Fig 4B). On visual quality control of such cases, we found that the scans contained substantial metal implants, interfering with the model’s ability to fully segment the spinal cord. It must be noted that all baselines were trained specifically for segmenting the spinal cord, whereas the proposed SCIseg 3D model can segment both the spinal cord and SCI lesions simultaneously.

***Lesion segmentation.— ***Table 3 shows a comparison between the 2D and 3D variants of the SCIseg model. The 3D model performed significantly better than the 2D model for both sites. As for the performance between sites, the 3D model’s performance on site 2 was higher than that of site 1 (see Table 3 for the *P* values). Through visual quality control, we found that site 1 contained several patients with metal implants causing heavy image artifacts. Additionally, SCI lesions spanned different phases (acute and subacute) with various degrees of lesion hyperintensity, thus making automatic segmentation challenging.

**Table 3:**
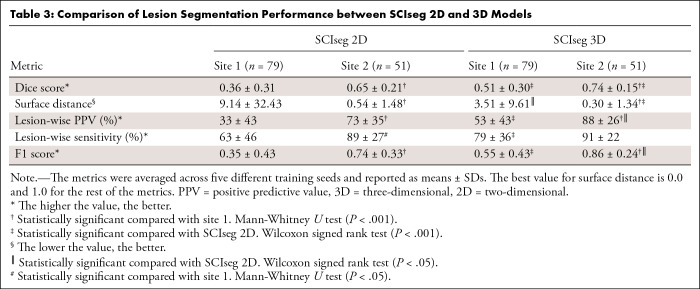
Comparison of Lesion Segmentation Performance between SCIseg 2D and 3D Models

### Effect of Active Learning on Lesion Segmentation

**Figure 5:**
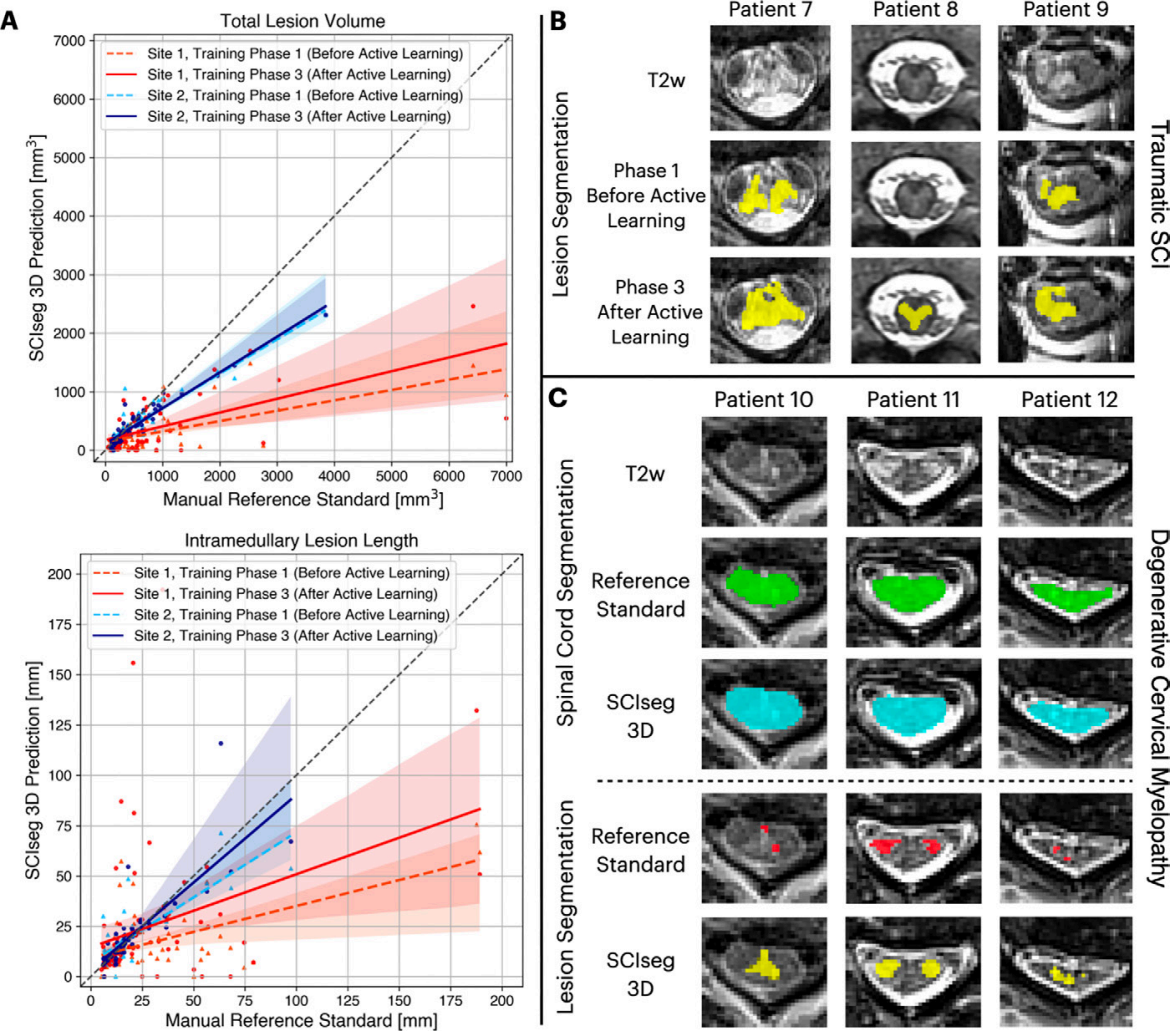
Comparison of model performance before and after active learning. **(A)** Correlation plots for total lesion volume (top) and intramedullary lesion length (bottom) computed from the manual reference standard lesion masks (x-axis) and lesion segmentation predictions from the proposed SCIseg 3D model (y-axis). Within each plot, colored dashed and solid lines show the agreement between the manual reference standard and automatic predictions before and after active learning, respectively, for site 1 (red and orange) and site 2 (blue and light blue). The model’s predictions after active learning show a higher agreement with the manual reference standard for both sites (ie, solid lines move closer to the diagonal identity line). **(B)** SCIseg’s predictions on unseen axial scans from site 2 before and after active learning. **(C)** Examples of SCIseg’s generalization to patients with nontraumatic spinal cord injury (ie, degenerative cervical myelopathy). The model obtains an accurate spinal cord segmentation even at the level of severe compression (patient 12). Green and teal colors refer to the reference standard and automatic predictions for the spinal cord. Red and yellow refer to the reference standard and the automatic predictions for lesions. 3D = three-dimensional, T2w = T2-weighted.

We performed an ablation study comparing the model performance after phase 1 (training on two sites) and phase 3 (training on three sites after active learning). Figure 5A shows the correlation between manual reference standard and automatic predictions for total lesion volume (top) and intramedullary lesion length (bottom). For both sites, higher agreement between the manually annotated and automatically derived lesion metrics was observed for the final model after the third phase of training (ie, solid lines moving closer to the diagonal identity line). There was a statistically significant improvement after active learning (phase 3) in estimating the total lesion volume (*P* = .016) and the lesion length (*P* = .004) for site 1.

Figure 5B shows the lesion segmentation performance of our baseline model after phase 1 of training (before active learning) on unseen axial T2-weighted scans from site 1. Qualitatively, the model tends to undersegment the lesions. However, there was an overall improvement in segmentation performance when the model was trained on more data consisting of axial scans from site 1 and isotropic sagittal scans from site 3 during phase 3 of training.

### Generalization to DCM

Qualitative examples of spinal cord and lesion segmentation on an independent dataset of patients with DCM whose data were unseen during model training are shown in Figure 5C. Interestingly, in cases in which the reference standard lesion masks were undersegmented, the model provided a better and more complete segmentation of the lesion. Furthermore, the spinal cord segmentations were accurate even for sections with severe spinal cord compression (Fig 5C, patient 12). Table 4 shows the Dice and F1 scores for both spinal cord and lesion segmentations. Despite not being trained on DCM lesions, the model achieved a high mean Dice score of 0.95 ± 0.01 for spinal cord segmentation and a mean F1 score of 0.49 ± 0.44 for lesion segmentation.

**Table 4:**
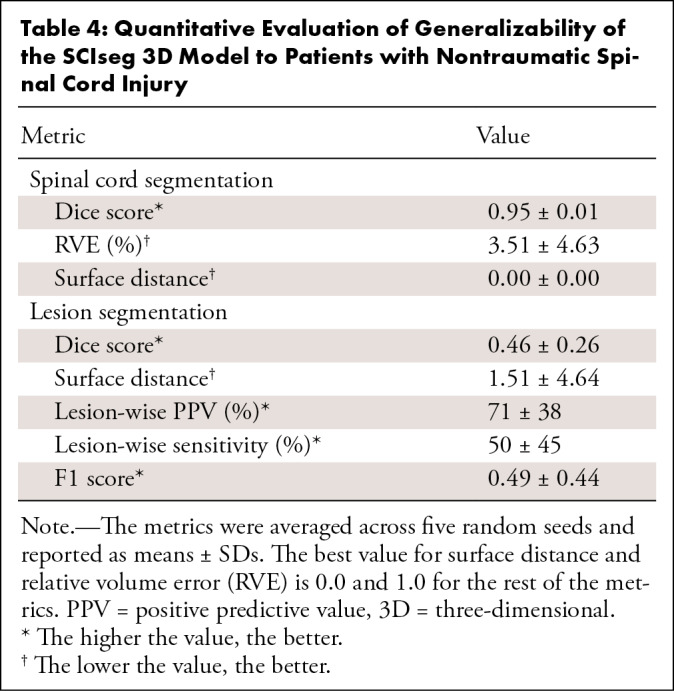
Quantitative Evaluation of Generalizability of the SCIseg 3D Model to Patients with Nontraumatic Spinal Cord Injury

### Manual versus SCIseg-predicted Lesion Biomarkers

**Figure 6:**
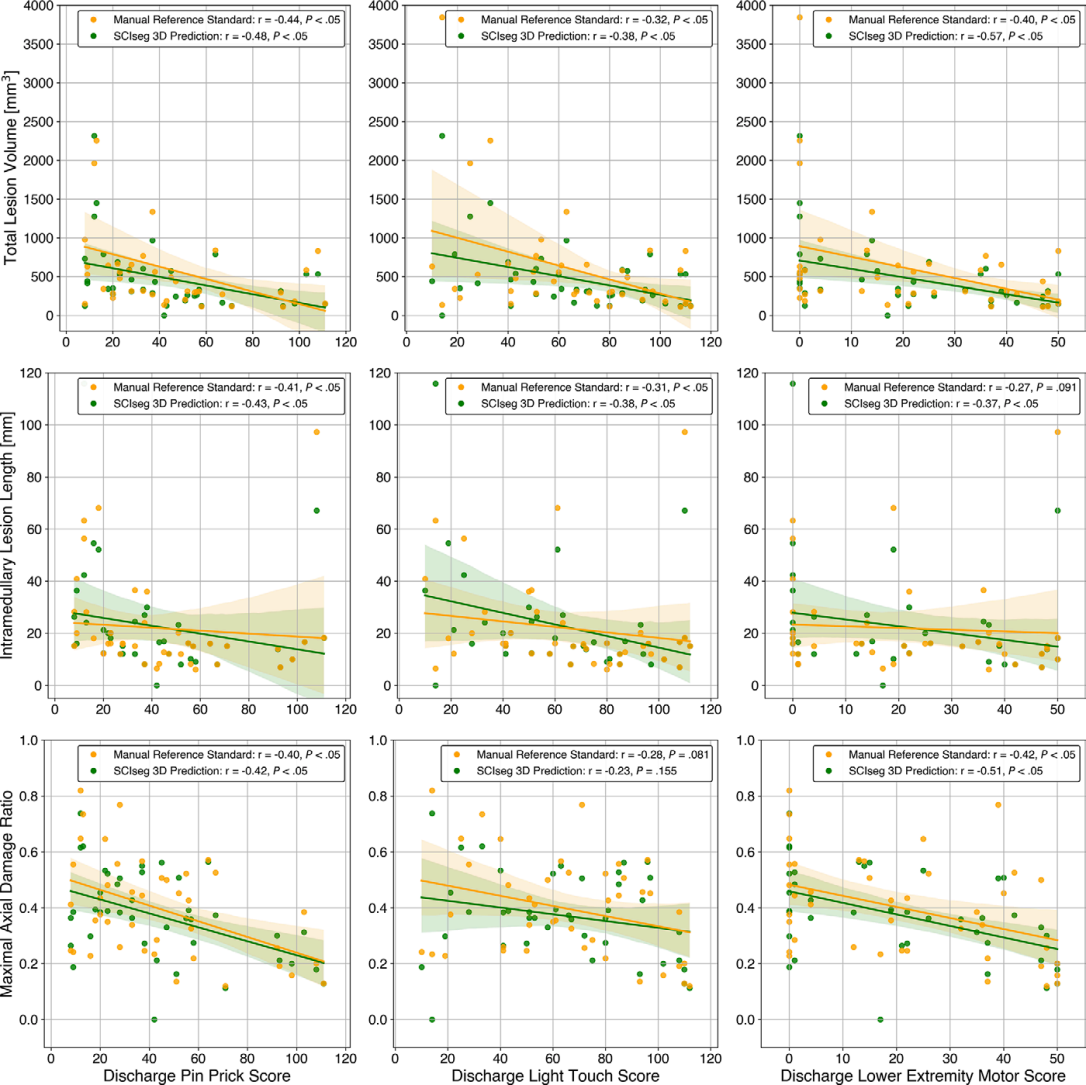
Correlation analysis between discharge clinical scores (x-axis) and quantitative MRI biomarkers (y-axis) for site 2. Spearman correlation coefficient and *P* value are shown in the legends of individual subplots. The Wilcoxon signed rank test between the manual reference standard lesion masks (yellow) versus automatic predictions using SCIseg 3D (green) lesion biomarkers revealed no evidence of differences for lesion length (*P* = .42) and maximal axial damage ratio (*P* = .16) but a significant difference for lesion volume (*P* = .003). 3D = three-dimensional.

Quantitative MRI biomarkers obtained from SCIseg 3D predictions were comparable with those obtained from manually segmented lesions (Fig 6). The Wilcoxon signed rank test between SCIseg-predicted (Fig 6, green) versus manual reference standard (Fig 6, yellow) lesion biomarkers revealed a significant difference in lesion volume (*P* = .003) between the two groups and no evidence of a difference for lesion length (*P* = .42) and maximal axial damage ratio (*P* = .16).

### Correlation between Clinical Scores and MRI Biomarkers

Figure 6 shows the correlation plots (including correlation coefficients and *P* values) between clinical scores and quantitative MRI biomarkers calculated from both manual reference standard lesion masks and lesions segmented using SCIseg 3D.

## Discussion

This study introduced a DL-based model, SCIseg, for the automatic segmentation of the spinal cord and intramedullary lesions in patients with SCI on T2-weighted MRI scans. The model was trained and evaluated on a cohort of 191 patients with traumatic and nontraumatic SCI with 231 scans acquired using different scanner manufacturers with heterogeneous image resolutions (isotropic and anisotropic) and orientations (axial and sagittal). Patients had various lesion etiologies (traumatic, ischemic, and hemorrhagic) and lesions spread across the cervical, thoracic, and lumbar spine. SCIseg achieved a mean Dice score of 0.92 ± 0.07 for spinal cord segmentation and 0.61 ± 0.27 for SCI lesion segmentation. There was no evidence of a difference between lesion length (*P* = .42) and maximal axial damage ratio (*P* = .16) computed from manually annotated lesions and the lesion segmentations obtained using SCIseg. To the best of our knowledge, SCIseg is the first open-source, automatic method for lesion and spinal cord segmentation in SCI. It also generalizes to patients with DCM, producing accurate segmentations for both lesions and the spinal cord at the compression levels.

As the segmentation performance might be constrained by the low data quality and small dataset sizes in SCI, we showed that implementing a three-phase training strategy, including an active learning approach to progressively expand the dataset size and incorporate diverse data distributions into the training set, enhanced the model’s performance. Furthermore, a region-based training strategy that jointly segments the spinal cord and the lesion was more efficient than training two individual models for spinal cord and lesion segmentation, respectively. As a result, correlation analyses between clinical scores and MRI-derived biomarkers showed statistically significant relationships for both manually annotated reference standard and automatically derived lesion masks, suggesting the SCIseg predictions can be reliably used for correlation with clinical measures in SCI.

Our cohort predominantly consisted of traumatic SCI lesions in intermediate and chronic phases, as the prevalence of ischemic and hemorrhagic lesions is typically lower (4). As chronic injuries tend to be more delineated on T2-weighted scans (2), our model learned to be sensitive to hyperintense abnormalities in the image. This sensitivity to hyperintense abnormalities also explains its ability to segment DCM lesions, which tend to be hyperintense at the site of compression. Similarly, as the injury levels in the training dataset were skewed toward the cervical spine, the model’s ability to segment lumbar lesions was expected to be lower when compared with cervical or thoracic lesions. Despite the presence of metal implants causing strong image artifacts in several patients, SCIseg provided a good starting point for obtaining lesion and spinal cord segmentations instead of manual annotations from scratch.

Only a few studies exist in the literature discussing the importance of automatic segmentation in SCI scans (24,36). The study by McCoy et al (24) is most similar to ours, as it presented the first DL method for segmentation of the spinal cord and intramedullary lesions in SCI. Nevertheless, there are several important distinctions between the two studies. Although their model was trained on axial preoperative scans of patients with acute SCI from a single site, our model was trained on multisite data consisting of patients with traumatic, ischemic, and hemorrhagic SCI with different image orientations (axial and sagittal). Moreover, our model was exposed to more heterogeneous data covering different injury phases (intermediate and chronic) and therefore demonstrated better generalization to both traumatic and nontraumatic lesion etiologies. More importantly, our work is open source, further enabling reproducible, multisite studies in SCI.

Several promising avenues for future work exist. The segmentation models can be improved by using more fine-grained reference standard masks, in which the hyperintense edema and hypointense hemorrhage could be treated as separate classes. Training a model on preoperative traumatic SCI data using these reference standard masks would have a major impact on improving the initial classification of the disease and further prognostication (18). Although the model generalized reasonably well to DCM lesions, there is potential for improvement, particularly by adding the DCM cohort to the existing training set or by training a DL model exclusively on DCM data. Previous studies have reported the presence of hyperintense T2-weighted lesions in up to 64% of patients with DCM (13,37,38) and explored the relationship between structural and functional damages (39). Such studies would greatly benefit from an automatic DCM lesion segmentation method. Aggregating more heterogeneous data will also allow for sensitivity analysis of potential confounding factors.

This study had limitations. First, longitudinal scans from patients with follow-up examinations were treated as independent inputs for training. Although the lesion appearance evolved between sessions, resulting in nonidentical lesions (hence justifying our choice of treating them as independent inputs), the model likely could not learn the evolution of lesions across time. Second, the model’s sensitivity to hyperintense abnormalities might result in false-positive segmentations in healthy controls where the spinal cord central canal is visualized. Third, our limited size of 196 MRI scans in the training set risks overfitting, given the complexity of the SCI lesion segmentation task. Although we gathered diverse data from three sites and trained five models on different train/test splits, along with extensive data augmentation to prevent potential overfitting, increasing the dataset size would further improve the model’s performance and generalization. Last, we did not analyze the interrater variability as the data were gathered from multiple sites and there were no overlapping patients across sites. Previous studies (40,41) have reported that MRI measures of spinal cord damage (eg, edema length, midsagittal tissue bridge ratio, axial damage ratio) do exhibit high to excellent levels of interrater reliability.

In conclusion, this study presented SCIseg, an automatic DL-based method, for the segmentation of the spinal cord and intramedullary lesions in SCI on T2-weighted MRI scans. The work has addressed several limitations of previous studies by including a large retrospective cohort consisting of 191 patients spanning three sites, using MRI data acquired with scanners from different manufacturers, and training a single model to segment both the spinal cord and SCI lesions. More importantly, the method has been designed to ensure reproducibility and enable large-scale, reproducible prospective studies. The model is open source and accessible via the Spinal Cord Toolbox (version 6.2 and higher). We hope that SCIseg will benefit clinicians and patients by providing additional diagnostic and prognostic information, serving as a basis for further studies assessing optimal rehabilitation from a customized patient-based perspective.
